# Margin weighted robust discriminant score for feature selection in imbalanced gene expression classification

**DOI:** 10.1371/journal.pone.0325147

**Published:** 2025-06-10

**Authors:** Sheema Gul, Dost Muhammad Khan, Saeed Aldahmani, Zardad Khan

**Affiliations:** 1 Department of Statistics, Abdul Wali Khan University, Mardan, Pakistan; 2 Department of Statistics and Business Analytics, United Arab Emirates University, Al Ain, United Arab Emirates; The University of Sheffield, UNITED KINGDOM OF GREAT BRITAIN AND NORTHERN IRELAND

## Abstract

High-dimensional gene expression data poses significant challenges for binary classification, particularly in the context of feature selection methods. Conventional methods, for example, Proportional Overlap Score, Wilcoxon Rank-Sum Test, Weighted Signal to Noise Ratio, ensemble Minimum Redundancy and Maximum Relevance, Fisher Score and Robust Weighted Score for unbalanced data are impacted by key challenges, such as, class imbalance and redundancy. To mitigate these issues, customized feature selection methods are required to tackle the class imbalance issue.

This study proposes a more robust solution, Margin Weighted Robust Discriminant Score, for feature selection in the context of high dimensional imbalanced problems. MW-RDS integrates a minority amplification factor to ensure the impact of minority class observation during feature ranking process. The amplification factor along with class specific stability weights obtained from minority-focused robust discriminant score are used for achieving maximum differential capability of genes/features. The score is weighted by margin weights extracted from support vectors to enhance the discriminative power of genes/features thereby highlighting its potential for class separation. Finally, top-ranked genes/features are constrained using $\ell_{1}$-regularization to discard redundant genes while identifying the most significant ones.

The performance of the proposed method is tested on 9 openly accessible gene expression datasets, using Random Forest, Support Vector Machines, and Weighted *k* Nearest Neighbors classifiers in term of performance metrics, i.e., accuracy, sensitivity, specificity, F1-score, and precision. The results reveal that the proposed method outperforms the existing methods in most of the cases. Boxplots and stability-plots are also generated to gain a deeper understanding of the results. To futher assess the efficacy of the proposed method, the paper also gives a detailed simulation study.

## 1 Introduction

The analysis of high dimensional gene expression data has a significant contribution in the growth of biomedical research, particularly, in biomarkers’ discovery and understanding molecular mechanisms. Derived from technologies such as DNA microarrays and RNA sequencing, high-dimensional datasets capture the simultaneous behaviours of thousands of genes. However, getting insights from these datasets is challenging due to the presence of many features that are noisy, redundant or irrelevant. Consequently, detecting genes that regulate the target class is difficult [1,2]. Moreover, skewed class distribution, i.e., fewer observations in one class than the other, in most of the gene expression datasets further complicates the process of identifying regulatory genes. This leads to a reduction in the efficiency of the models trying to identify hidden patterns in the minority class instances – desired in many clinical applications. Many of the classical methods in the field of machine learning [3] are tailored to yield high predictive performance for the majority classes while overlooking the minority classes which carry significant importance in diagnostic and predictive analyses. The problems of high dimensionality and skewed class distribution are especially acute in computational biology where models may become tuned to the class with majority observations and thereby poorly identify the class with fewer instances [4–7]. These issues highlight the need of algorithms that can effectively handle high dimensionality and class imbalance problems simultaneously. To this end, feature selection is often used in selecting genes with high regulatory power and discard noisy and redundant ones to increase the accuracy and interpretability, while maintaining optimal computational cost [8,9].

Feature selection methods are generally classified into three categories, i.e., wrapper, filter, and embedded. Wrapper methods assess subsets of features using a specific algorithm to achieve optimized model performance. Examples of wrapper methods [10–12] are forward selection, backward elimination, and recursive feature elimination (RFE) methods. Embedded feature selection methods [13] combine feature selection with the model training process including LASSO, ridge regression, and decision tree. Filter methods [14] rank features, as a pre-processing step, based on statistical significance. Examples include Pearson correlation coefficient, Relief based algorithm, and Minimum Redundancy Maximum Relevance (MRMR) method. Most of these methods treat both minority and majority classes equally ignoring the adverse effect of the class imbalance problem [15]. Many of the traditional feature selection methods [16,17] give poor performance in class imbalance scenarios. Several efforts have been made in the literature to tailor these methods for class imbalance [18]. However, these methods have been found to struggle with achieving scalability and robustness [19–21].

Inspired from the above-mentioned notion, the current article suggests a feature selection method, the Margin Weighted Robust Discriminant Score (MW-RDS), tailored for high-dimensional gene expression dataset with class imbalance problem. The method consists of ranking genes based on their differential capability between two classes using a minority amplification factor that guarantees adequate representation of the minority class observation. The scores are weighted by margin weights obtained from support vectors for achieving maximum differential capability leading to an overall minority-focused robust discriminant score (RDS). Top ranked genes are further penalized via $\ell_{1}$-regularization to eliminate redundant genes while selecting statistically and biologically relevant ones.

The proposed MW-RDS is assessed on a total of 9 benchmark gene expression datasets using Random Forest (RF), support vector machines (SVM), and weighted *k*-nearest neighbors (W*k*NN) classifiers. Classification accuracy, specificity, F1-score, sensitivity, and precision are used as performance metrics. The results are compared with those of the traditional methods such as Proportional Overlap Score (POS), Wilcoxon Rank-Sum Test (Wilcoxon), Weighted Signal-to-Noise Ratio (WSNR), ensemble Minimum Redundancy and Maximum Relevance (mRMRe), Fisher Score (Fisher) and robust weighted score for unbalanced data (ROWSU). A detailed simulation study, demonstrating class imbalance problem, is also given.

The remainder of this paper is arranged as follows: Sect 2 gives a thorough review of the related work. A complete description of the suggested MW-RDS method is given in Sect 3. The experimental design and results based on the benchmark and contrived datasets are given in Sect 4, while Sect 5 concludes the findings of this work.

## 2 Related work

In the literature, several methods have been proposed for feature/gene selection in high dimensional gene expression datasets with class imbalance problem. Due to this problem, patterns related to the minority class are often overlooked in that feature selection/classification methods mostly learn from the patterns of majority class observations. Therefore, feature selection plays significant role in identifying genes/features that are most relevant to a specfic classification and results in improved model with minimum complexity [22–24]. Several of these methods are prioritizing overall data trends often neglecting class imbalance problem and redundancy. Addressing these problems requires customized feature selection methods that achieve equity in the representation of minority class and improve predictive accuracy [25–29].

Some existing methods, such as robust masking technique [30], addresses this issue to some extent by minimizing noise and outlier. These method are efficient for handling expression outliers, however, they perform inadequately in dealing with class imbalance problems constraining their relevance in high dimensional datasets. Methods like minimum redundancy maximum relevance (mRMR) along with its extension, i.e., minimum redundancy maximum relevance ensemble (mRMRe) has shown improved results in high dimensional problems. These methods achieve maximum relevance with target variable while reducing redundancy [31–33]. However, these methods face computational challenges and lack a direct mechanism to prioritize minority class problems [34].

Other statistical approaches, such as the Wilcoxon Rank-Sum Test [35] and Fisher Score [36], have been valuable for assessing feature relevance but are often limited by their assumption of balanced datasets. Weighted adaptations of these methods attempt to address the class imbalance [37], yet they frequently overlook feature interactions and correlations, resulting in suboptimal feature subsets [38]. Techniques such as Weighted Signal-to-Noise Ratio (WSNR) prioritize features based on their contribution to class distinctions [39], but their reliance on precise signal estimation makes them susceptible to noise [40]. Decision tree-based methods, like Boruta, offer effective feature ranking while addressing class imbalance [41], but their computational intensity can be a barrier for large-scale datasets [42]. Similarly, evolutionary algorithms and embedded approaches, such as Sparse Autoencoders, have shown promising results in addressing class imbalance but are often constrained by high computational costs due to their iterative nature [20,43,44]. While significant advancements have been made, existing methods still face challenges in fully addressing minority class, especially when dealing with noisy, high dimensional imbalance problems.

Considering the above challenges associated with high dimensional class imbalanced problems, a customized feature selection method has been proposed. This method highlights the importance of minority class in feature scoring process and proposes minority-focused robust discriminant score (RDS) with class-specific stability weights to focus on biologically significant features. These selected gene/features are further refined by separating classes through margin weights obtained from support vectors and to remove redundancy by $\ell_{1}$-regularization resulting in a concise and discriminant feature set. The proposed method has been effective in addressing class imbalanced problems thus offers a promising strategy where the existing methods often face challenges with skewed distributions.

## 3 Margin Weighted Robust Discriminant Score (MW-RDS)

Let the gene expression dataset be expressed as 𝒵=(𝒳,𝒴), where 𝒳 is the feature matrix consisting of κ samples and *p* features, defined as:

$$𝒳=[x_{ab}{]}_{\kappa \times p}\in {\mathbb{R}}^{\kappa \times p},$$ (1)

where, a=1,2,3,...,κ and b=1,2,3....,p. The binary class variable is given as, $𝒴\in \{-,+{\}}^{\kappa }$.

The dataset is divided in to two groups, i.e., ${𝒳}^{+}\in {\mathbb{R}}^{\kappa^{+}\times p}$ and ${𝒳}^{-}\in {\mathbb{R}}^{\kappa^{-}\times p}$ representing the feature matrix of minority and majority class observations, respectively. The symbol *p* represents the number of features and $\kappa^{+}$ and $\kappa^{-}$ represent the number of minority and majority class observations, respectively. To mitigate the effects of class imbalance, a minority implification factor (τ) that quantifies the degree of imbalance is introduced. Mathematically,

$$\tau =\frac{\left|\kappa^{+}\right|}{\left|\kappa^{-}\right|}.$$ (2)

Since the class distribution is highly imbalanced i.e., $\left|\kappa^{-}|\gg |\kappa^{+}\right|$, τ act as a minority amplification factor that is used to balance the influence of minority class during feature scoring process. Particularly, the contribution of minority class feature matrix ${𝒳}^{+}$ compared to majority class features is amplified by τ given in 2. This amplification factor ensures the discrimination efficacy of genes relevant to the minority class within the feature scoring process to prevent the dominant relevance of the majority class. The proposed method adjusts the influence of the minority class and gives slightly more weight to the minority class using a factor of (1  +  τ). This ensures that class-specific features are effectively captured, preventing their loss due to skewed data. Using the amplification factor τ, a robust discriminant score (RDS) is introduced that combines amplification factor (τ) and further assigns importance to genes/features that effectively differentiate the minority class improving the overall robustness of the model.

### 3.1 Minority-focused robust discriminant score

Once the factor τ is computed, it is used to fine-tune the robust discriminant score (RDS), enabling a tailored adjustment that specifically targets class imbalance. Minority-Focused robust discriminant score (RDS) uses class specific stability weights to select genes that show high stability within majority and minority classes. These weights are inversely proportional to the variance of the *b*^*th*^ gene. The weights can be expressed as follows:

$$\upsilon_{b}^{+}=(\text{V}(\eta_{b}^{+}){)}^{-1},\;\upsilon_{b}^{-}=(\text{V}(\eta_{b}^{-}){)}^{-1},$$ (3)

where, $\eta_{b}^{+}$ and $\eta_{b}^{+}$ signify the medians of the *b*^*th*^ gene for minority and majority classes, respectively. Using these weights, the robust discriminant score ($\Phi_{b}$) is computed as:

$$\Phi_{b}=\frac{\upsilon_{b}^{+}(1+\tau )|\eta_{b}^{+}-\zeta_{b}|+\upsilon_{b}^{-}|\eta_{b}^{-}-\zeta_{b}|}{\upsilon_{b}^{+}(1+\tau )\theta_{b}^{+}+\upsilon_{b}^{-}\theta_{b}^{-}},\;\text{where }b=1,2,3,\ldots ,p$$ (4)

In the above expression, the term $\zeta_{b}$ is the combined median of the *b*^*th*^ gene/feature across all observations, and $\theta_{b}^{+}$ and $\theta_{b}^{-}$ indicate the mean absolute deviations of the *b*^*th*^ gene/feature for the minority and majority class observations, respectively. Thus, the score given in Eq 4 is synthesized by combining key terms, i.e., minority amplification factor given in Eq 2 and class specific stability weights given in Eq 3. The resulting score, referred to as Φ given in Eq 5 is formulated as a sequence of discriminant scores for ranking genes based on their differential capability within classes, i.e.,

$$\Phi =\{\Phi_{1},\Phi_{2},\Phi_{3},\ldots ,\Phi_{p}\}.$$ (5)

### 3.2 Margin-weighted feature scoring

For further increase in the differential capability of the above scores ($\Phi_{b}$), they are weighted by weights, say γ, derived from support vectors, i.e., $D^{*}=\{(x_{a},y_{a})\in 𝒳|\gamma_{a}\geq 0\}$. For any feature *b*, its margin weights is defined as $w_{b}=|w_{b}|$, this method quatifies how much the *b*th feature leads to better class distinction, facilitating the ranking of more discriminative features. This maximizes the margin between the hyperplane and data observations. The weights derived from support vectors can briefly be summarized by the following expressions.

$$\omega =\sum_{a=1}^{\kappa^{*}}\gamma_{a}y_{a}x_{a},\;\text{where }a=1,2,3,\ldots ,\kappa^{*}$$ (6)

$$\omega =\{\omega_{1},\omega_{2},\omega_{3},\ldots ,\omega_{p}\},$$ (7)

where, $x_{a}\in {\mathbb{R}}^{p},a=1,2,3,\ldots ,\kappa^{*}$, stands for the the feature vector of the *a*_*th*_ support vector, with *a* indexing each support vector and $\kappa^{*}$ representing the total number of support vectors.

Let $y_{a}\in \{-,+\}$ represents the class label for the *a*^*th*^ observation. The term $\gamma_{a}$ represents the dual coefficient associated with the support vectors *x*_*a*_. For each feature, these coefficients are employed to calculate the absolute margin weight, indicating the degree to which a feature affects the classification boundary through the support vectors. The vector of weights, ω, shown in Eq 7 helps in identifying gene/feature that contribute in separating the classes. The final robust score $\Psi_{b}$ for each gene/feature is estimated as,

$$\Psi_{b}=|\Phi_{b}\cdot \omega_{b}|,\;\text{where }b=1,2,3,\ldots ,\hat{p}$$ (8)

The robust score, $\Psi_{b}$ of the *b*th gene/feature, represents its combined strength to differentiate between the two classes.

### 3.3 Feature ranking and redundancy elimination

While features are ranked based on their final robust score $\Psi_{b}$, high-dimensional imbalanced problems often contain redundant features. To further refine the selection of informative genes/features, redundancy problem is addressed through least absolute shrinkage and selection operator [45], an $\ell_{1}$-regularization that promotes sparsity. The corresponding optimization problem is:

$$\hat{\beta }=\arg\min_{\beta }\;ℒ(\beta ;{𝒢}_{b},y_{b})+\lambda \|\beta {\|}_{1}.$$ (9)

The function, denoted by $ℒ(\beta ;{𝒢}_{b},y_{b})$, is used for the loss function over the feature subset. Here, ${𝒢}_{b}\in {\mathbb{R}}^{\kappa \times d}$, where d≪p and λ>0 regulates the degree of sparsity during optimization. The set ${𝒢}_{b}$ contains the *d* top ranked features based on the scores computed by $\Psi_{b}$. For binary classification, the loss is defined as the negative log-likelihood of the logistic regression model. This regularization framework is embedded within logistic regression to optimize the feature selection process by minimizing the following penalized objective function:

$$ℒ(\beta )=-\sum_{a=1}^{\kappa }\left[y_{a}\log(d_{a})+(1-y_{a})\log(1-d_{a})\right]+\lambda \sum_{b=1}^{d}|\beta_{b}|,$$ (10)

where $d_{a}=\frac{1}{1+e^{-{𝐱}_{a}^{⊤}\beta }}$ denotes the logistic link function, mapping linear combinations of features to probabilities.

The parameter λ>0 controls the trade-off between model fit and sparsity, while the term $\sum_{b=1}^{d}|\beta_{b}|$ indicates $\ell_{1}$-regularization that shrinks redundant features’ coefficients towards zero.

Consequently, the final set of genes is given as:

$${𝒢}^{*}=\{\text{Top-ranked features by}\;{𝒢}_{b}\}\cap \{\text{features with }\beta_{j}\neq 0\text{ after }e_{1}\text{-regularization}\}.$$ (11)

The final feature set, denoted as, ${𝒢}^{*}\subset {𝒢}_{b}$ given in Eq 1 includes the top ranked discriminative features. The combination of robustness, margin-based significance, and sparsity results in a final gene/feature set that effectively balances clarity and performance, especially in high-dimensional, imbalanced contexts like gene expression analysis.

**Fig 1:**
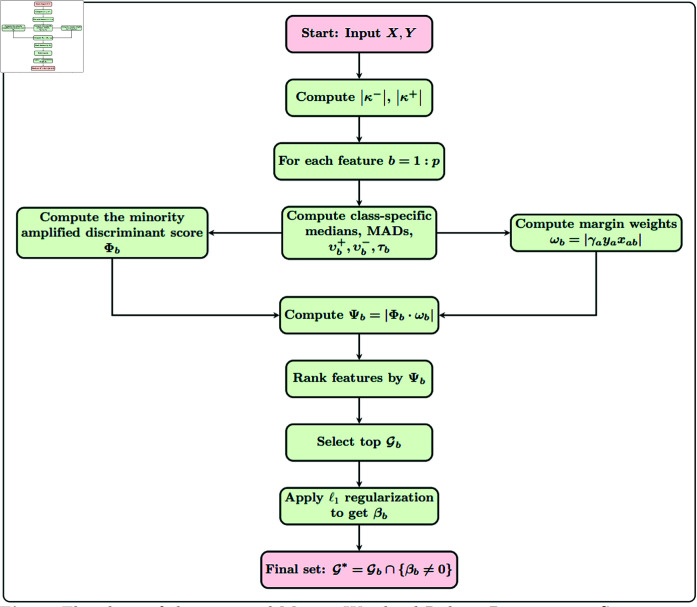
Flowchart of the proposed Margin Weighted Robust Discriminant Score (MW-RDS) algorithm.

The Algorithm detailed in 1 contains the pseudo-code of the proposed method, MW-RDS, and its corresponding flowchart is provided in Fig 1. The proposed MW-RDS algorithm begins with the computation of a robust score, followed by margin based weighting using support vectors and then ranking the features along with penalizing redundand features via the $\ell_{1}$ penalty.

**Algorithm 1. Margin Weighted Robust Discriminant Score (MW-RDS).**



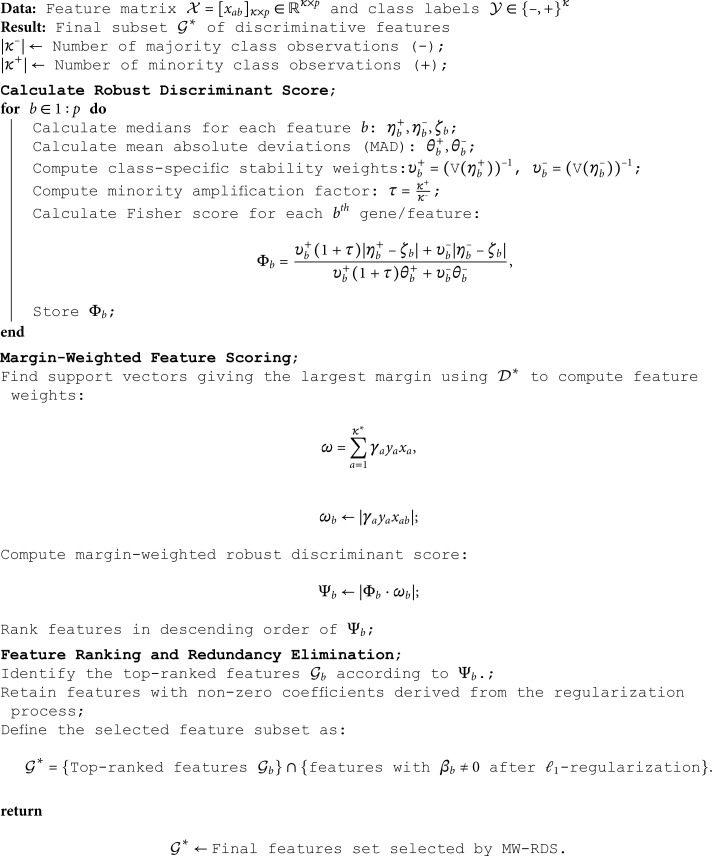



## 4 Experiments and results

This section outlines the experimental design, analysis on benchmark and simulated dataset, and the evaluation metrics. Detailed explanations of the experimental setup and the study’s findings are provided in the following subsections.

### 4.1 Imbalanced gene expression datasets

To assess how well the proposed method performs compared to the existing methods, we used nine benchmark imbalanced gene expression datasets. A quick overview of these datasets is provided in Table 1. In the give table, the first column lists the dataset ID, followed by the dataset name in the second column. The third and fourth columns show the number of observations (κ) and features (*p*), respectively. The fifth column gives the class-wise distribution (negative and positive classes), and the last column provides the data sources.

**Table 1:** Summary of the gene expression datasets. Number of samples, number of features, and class-wise frequency distribution are shown against each dataset.

ID	Data Name	κ	*p*	Class Distribution	Source
*ID*_1_	Endometrium Uterus	155	10936	31/124	https://www.openml.org/search?type=datastatus=activeid=1164
*ID*_2_	Prostate	89	10936	20/69	https://www.openml.org/search?type=datastatus=anyid=1141
*ID*_3_	Kidney	337	10936	77/260	https://www.openml.org/d/1147
*ID*_4_	Leukaemia	72	7129	23/49	https://rdrr.io/cran/propOverlap/man/leukaemia.html
*ID*_5_	Breast Colon	402	10936	58/344	https://www.openml.org/d/1458
*ID*_6_	Colon	62	2000	22/40	https://www.openml.org/d/1458
*ID*_7_	Ovary Lung	223	10936	25/198	https://www.openml.org/search?type=datastatus=activeid=1140
*ID*_8_	Breast	59	4870	15/44	https://openml.org/search?type=datastatus=activeid=45085
*ID*_9_	Ova Endometrium	801	10936	61/800	https://www.openml.org/search?type=datastatus=activeid=1142

### 4.2 Experimental setup

As the focus of this study is high dimensional imbalanced data classification, the given data were adjusted to maintain a 9:1 ratio, with 90% of the observations in the negative class and 10% in the positive class. This is done by randomly removing the minority class observation to create the 9:1 imbalance ratio. For a fair validatioin purpose, each dataset is split into 70% training data, used for building models, and 30% testing data, used to evaluate the methods’ performance. A total of 500 runs of the split sample estimates are obtained to validate the findings and assess the performance of proposed method against the other competitors. The 500 split-sample runs were performed for ensuring the robustness and reliability of the evaluation, allowing for the assessment of performance variability under different data partitions. This extensive validation gives a comprehensive and fair comparison between the MW-RDS approach and competing methods, controling the influence bias in random sampling. It is worth emphasizing that, in both feature selection and classifier application on the selected features, the same training and testing sets are used for all the methods under each run of the experiments.

Using the above setup, top 10 genes are selected by the proposed MW-RDS method and the other feature selection methods, i.e., Proportional Overlap Score (POS), Wilcoxon Rank-Sum Test (Wilcoxon), Weighted Signal-to-Noise Ratio (WSNR), ensemble Minimum Redundancy and Maximum Relevance (mRMRe), Fisher Score (Fisher) and robust weighted score for unbalanced data (ROWSU). Classification model, i.e., random forest (RF), support vector machine (SVM) and weighted *k nearest neighbors* (*Wk*NN) were used to evaluate the efficacy of the selected features, using performance metrics, i.e., accuracy, sensitivity, specificity, F1-score and precision. To guarantee the accuracy and reproducibility, the experiments were carefully conducted in R programming. All three classifiers are used using their default values of the hyper parameters as given in the corresponding R packages.

### 4.3 Results

This section provide the results of the proposed method, i.e., MW-RDS and other competing feature selection methods, i.e., POS, Wilcoxon, WSNR, mRMRe, Fisher and ROWSU applied to the nine datasets, *ID*_1_, *ID*_2_, *ID*_3_, *ID*_4_, *ID*_5_, *ID*_6_, *ID*_7_, *ID*_8_ and *ID*_9_.

Table 2 reveals a detailed comparison of feature selection methods, i.e., POS, Wilc, WSNR, mRMRe, Fisher and MW-RDS using different classification models, i.e., RF, SVM and W*K*NN in term of accuracy, sensitivity, specificity, F1-score, and precision on *ID*_1_. The proposed method is performing efficiently throughout the analysis. MW-RDS, using RF, achieved the highest specificity and precision, that is, 0.9877 and 0.9928 respectively, among all the feature selection methods. Random forest in term of sensitivity for MW-RDS is also exceptionally high with a value of 0.9960. Accuracy and F1-score with the values 0.9766 and 0.9905, respectively, are the highest among all the methods, making MW-RDS a clear winner in the case of RF classifier. Methods like POS and WSNR significantly under-perform, with accuracy values of 0.7879 and 0.7736, respectively. While mRMRe comes close in some metrics, i.e., precision observed as 0.9526, it doesn’t achieve the same level of consistency across all the performance metrics as MW-RDS. SVM paired with MW-RDS delivers excellent performance. The accuracy at 0.9962 and precision at 0.9942 are the highest across all feature selection methods, and F1 Score at 0.9900 further highlights its balanced performance. Compared to other methods, MW-RDS outperforms POS, WSNR, and Fisher, which have notably lower specificity observed as 0.1059 for POS and 0.1883 for WSNR. While mRMRe achieves slightly higher sensitivity at 0.9982, MW-RDS provides a more consistent balance across all metrics, making it the most reliable choice for SVM. For WKNN, MW-RDS shows the best performance with the highest specificity of 0.3648 among all the feature selection methods, which is significantly better than the alternatives, such as, POS at 0.0246 and WSNR at 0.1933. Other methods, such as Fisher and Wilcoxon, perform poorly in terms of specificity at 0.1085 and accuracy at 0.7696, emphasizing the superiority of MW-RDS.

**Table 2:** Using the *ID*_1_ dataset, results of the 3 classifiers for the given feature selection methods.

Models	Metrices	POS	Wilc	WSNR	mRMRe	Fisher	ROWSU	MW-RDS
RF	Accuracy	0.9837	0.9859	0.9744	**0.9959**	0.8841	0.9940	0.9863
	Sensitivity	0.9870	0.9853	0.9819	0.9966	0.9530	0.9887	**1.0000**
	Specificity	0.9762	0.9892	0.9545	0.9933	0.6628	**0.9995**	0.9401
	F1 Score	0.9894	0.9907	0.9835	**0.9974**	0.9270	0.9939	0.9911
	Precision	0.9921	0.9966	0.9862	0.9982	0.9066	**0.9995**	0.9987
SVM	Accuracy	0.9652	0.9833	0.9767	0.9893	0.8326	0.9880	**0.9959**
	Sensitivity	0.9840	0.9954	0.9789	0.9884	0.9607	0.9814	**0.9967**
	Specificity	0.9063	0.9499	0.9698	0.9921	0.4310	0.9952	**0.9959**
	F1 Score	0.9777	0.9890	0.9849	0.9929	0.8986	0.9875	**0.9972**
	Precision	0.9729	0.9833	0.9916	0.9978	0.8513	0.9954	**0.9979**
W*k*NN	Accuracy	0.9878	0.9933	0.9778	**0.9989**	0.8504	0.9988	0.9900
	Sensitivity	0.9973	0.9916	0.9855	0.9995	0.9622	0.9977	**1.0000**
	Specificity	0.9539	**1.0000**	0.9517	0.9967	0.4775	**1.0000**	0.9648
	F1 Score	0.9922	0.9778	0.9858	0.9995	0.9080	**0.9988**	0.9998
	Precision	0.9874	**1.0000**	0.9867	**1.0000**	0.8636	**1.0000**	0.9869

Table 3 gives the results for *ID*_2_ dataset. MW-RDS using RF gives the highest sensitivity, F1-score and precision values, i.e., 1, 0.9401, 0.9911 and 0.9916, respectively. However, its accuracy is slightly lower than that of mRMRe that is 0.9959. Similarly, SVM paired with MW-RDS excels with the highest accuracy, perfect sensitivity, near-perfect specificity, and the highest F1-score that is 0.9959, 1, 0.9995 and 0.9972, respectively, making it the best-performing combination overall.

**Table 3:** Using the *ID*_2_ dataset, results of the 3 classifiers for the given feature selection methods.

Models	Metrices	POS	Wilc	WSNR	mRMRe	Fisher	ROWSU	MW-RDS
RF	Accuracy	0.7879	0.8215	0.7736	0.9481	0.7768	0.9230	**0.9766**
	Sensitivity	0.9814	0.9550	0.9144	0.9836	0.9681	0.9343	**0.9960**
	Specificity	0.0349	0.3278	0.1929	0.8123	0.0256	0.9125	**0.9877**
	F1 Score	0.8798	0.8941	0.8662	0.9675	0.8728	0.9224	**0.9908**
	Precision	0.7991	0.8437	0.8261	0.9526	0.7971	0.9125	**0.9928**
SVM	Accuracy	0.7934	0.7998	0.7687	0.8940	0.7966	0.9293	**0.9962**
	Sensitivity	0.9958	0.9502	0.9098	0.9812	**0.9982**	0.9608	0.9879
	Specificity	0.1059	0.2472	0.1888	0.5510	0.1085	0.8973	**0.9806**
	F1 Score	0.8840	0.8822	0.8632	0.9360	0.8859	0.9305	**0.9963**
	Precision	0.7962	0.8280	0.8253	0.8961	0.7978	0.9032	**0.9942**
W*k*NN	Accuracy	0.7706	0.8096	0.7651	**0.9319**	0.7426	0.6265	0.8074
	Sensitivity	0.9615	0.8853	0.8922	0.9749	0.9077	0.2505	**1.0000**
	Specificity	0.0246	0.5319	0.2380	0.7616	0.1085	**0.9994**	0.3648
v	F1 Score	0.8688	0.9074	0.8590	**0.9449**	0.8477	0.3959	0.8838
	Precision	0.7945	0.8919	0.8316	0.9730	0.8005	**0.9979**	0.9967

In the case of W*K*NN, MW-RDS demonstrates high Accuracy (0.9868), F1 Score (0.9938), and near-perfect Sensitivity (0.9995), showcasing its effectiveness. While mRMRe slightly outperforms MW-RDS in accuracy 0.9988 and precision, MW-RDS offers better balance across all metrics. Compared to other feature selection methods, MW-RDS consistently outperforms the others in terms of Sensitivity, often achieving perfect values for RF and SVM, and performs exceptionally well in terms of F1-score due to its balanced approach to precision and sensitivity. Although methods like Wilcoxon sometimes achieve higher specificity under W*K*NN with a value of 1, MW-RDS provides a more stable and consistent overall performance. Among the models, SVM paired with MW-RDS emerges as the best, delivering the best results across all the metrics, while W*K*NN is also competitive, particularly in sensitivity and F1-score. RF performs well but lags slightly in accuracy and precision. Overall, MW-RDS proves to be a reliable, high-performing feature selection method, standing out as a strong candidate for machine learning tasks in the presence of class imbalance.

Based on the results given in Tables 4, 5, 6, 7, 8, 9, and 10, similar conclusions can be drawn for the *ID*_3_ to *ID*_9_ datasets, where MW-RDS continues to demonstrate its strong and consistent performance across all the metrics.

**Table 4:** Using the *ID*_3_ dataset, results of the 3 classifiers for the given feature selection methods.

Models	Metrices	POS	Wilc	WSNR	mRMRe	Fisher	ROWSU	MW-RDS
RF	Accuracy	0.9917	0.9707	0.9783	**0.9921**	0.9412	0.9879	0.9859
	Sensitivity	0.9933	0.9783	0.9965	**0.9965**	0.9620	0.9805	0.9820
	Specificity	0.9861	0.9471	0.7583	0.9779	0.8696	0.9951	**0.9997**
	F1-Score	0.9946	0.9808	0.9883	**0.9948**	0.9620	0.9875	0.9908
	Precision	0.9960	0.9836	0.9804	0.9932	0.9620	0.9948	**1.0000**
SVM	Accuracy	0.9802	0.9693	0.9680	0.9836	0.9020	0.9842	**0.9837**
	Sensitivity	0.9921	0.9755	0.9760	0.9821	0.9620	0.9882	**0.9940**
	Specificity	0.9394	0.9508	0.8618	**0.9888**	0.6957	0.9707	0.9582
	F1-Score	0.9872	0.9799	0.9825	0.9892	0.9383	0.9842	**0.9895**
	Precision	0.9824	0.9845	0.9892	**0.9965**	0.9157	0.9707	0.9595
W*k*NN	Accuracy	0.9861	0.9716	0.9733	0.9896	0.8922	0.9855	**0.9999**
	Sensitivity	0.9917	0.9690	0.9917	**0.9965**	0.9367	0.9706	0.9855
	Specificity	0.9669	0.9819	0.7490	0.9678	0.7391	0.9844	**1.0000**
	F1-Score	0.9909	0.9833	0.9856	0.9950	0.9308	0.9849	**0.9999**
	Precision	**1.0000**	0.9868	0.9798	**1.0000**	0.9250	**1.0000**	0.9722

**Table 5:** Using the *ID*_4_ dataset, results of the 3 classifiers for the given feature selection methods.

Models	Metrices	POS	Wilc	WSNR	mRMRe	Fisher	ROWSU	MW-RDS
RF	Accuracy	**1.0000**	0.9691	0.9955	0.8227	0.9841	0.9406	0.9895
	Sensitivity	**1.0000**	0.9433	0.9989	0.7751	0.9903	**1.0000**	0.9763
	Specificity	**1.0000**	0.9836	0.9942	0.8557	0.9833	0.8807	0.9952
	F1-Score	**1.0000**	0.9500	0.9926	0.7211	0.9762	0.9440	0.9830
	Precision	**1.0000**	0.9655	0.9878	0.8889	0.9682	0.8970	**1.0000**
SVM	Accuracy	0.9018	0.9391	0.9641	0.8255	0.9373	0.9086	**0.9782**
	Sensitivity	0.9108	0.8824	0.9636	0.8888	0.8778	**0.9994**	0.9622
	Specificity	0.9033	0.9672	0.9678	0.8101	0.9721	0.8178	**0.9850**
	F1-Score	0.8535	0.8983	0.9417	0.7528	0.8965	0.9158	**0.9672**
	Precision	0.8229	0.9298	0.9304	0.9000	0.9418	0.8489	**1.0000**
W*k*NN	Accuracy	0.9036	0.9277	0.9659	0.805	0.9523	0.8810	**0.9700**
	Sensitivity	0.8696	0.8663	0.9781	0.6898	0.9488	**1.0000**	0.9916
	Specificity	0.924	0.9577	**0.9623**	0.8651	0.9584	0.7590	0.9601
	F1-Score	0.8471	0.8812	0.9454	0.6764	0.9262	0.8930	**0.9529**
	Precision	0.8000	**1.0000**	0.9224	0.8889	0.9186	0.8098	**1.0000**

**Table 6:** Using the *ID*_5_ dataset, results of the 3 classifiers for the given feature selection methods.

Models	Metrices	POS	Wilcoxon	WSNR	mRMRe	Fish	ROWSU	MW-RDS
RF	Accuracy	0.9789	0.9729	0.9746	**0.9915**	0.8627	0.9892	0.9868
	Sensitivity	0.9060	0.8796	0.8887	0.9606	0.1737	0.9125	**0.9794**
	Specificity	0.9911	0.9892	0.9892	**0.9970**	0.9826	0.9922	0.9960
	F1-Score	0.9230	0.9002	0.9087	0.9705	0.2708	**0.9892**	0.8897
	Precision	0.9447	0.9302	0.9356	0.9825	0.6138	0.9151	**0.9873**
SVM	Accuracy	0.9667	0.9686	0.9715	0.9823	0.8553	0.9847	**0.9886**
	Sensitivity	0.8475	0.8466	0.8968	0.9565	0.0856	**0.9772**	0.9042
	Specificity	0.9874	0.9895	0.9840	0.9869	0.9886	0.9022	**0.9912**
	F1-Score	0.8784	0.8819	0.8998	0.9408	0.1362	**0.9846**	0.9011
	Precision	0.9205	0.9295	0.9078	0.9287	0.4927	0.9197	**0.9437**
W*k*NN	Accuracy	0.9745	0.9717	0.9713	**0.9910**	0.8522	0.9261	0.9747
	Sensitivity	0.9127	0.8978	0.8780	0.9591	0.2809	0.8865	**0.9797**
	Specificity	0.9853	0.9844	0.9872	0.9966	0.9519	0.8519	**0.9971**
	F1-Score	0.9110	0.8910	0.8970	0.9632	0.3470	0.9312	**0.9739**
	Precision	0.9147	0.9375	0.9233	0.9524	0.5060	0.8717	**0.9580**

**Table 7:** Using the *ID*_6_ dataset,results of the 3 classifiers for the given feature selection methods.

Models	Metrices	POS	Wilc	WSNR	mRMRe	Fisher	ROWSU	MW-RDS
RF	Accuracy	0.9595	0.8637	0.8447	**0.9689**	0.7189	0.8925	0.8711
	Sensitivity	0.9618	0.8959	0.8864	0.9797	0.8563	0.8655	**0.9917**
	Specificity	**0.9552**	0.8140	0.7755	0.9500	0.5289	0.9181	0.8485
	F1-Score	0.9657	0.8906	0.8785	0.9752	0.7889	0.8870	**0.9796**
	Precision	0.9725	0.8959	0.8814	**0.9728**	0.7531	0.9173	0.8921
SVM	Accuracy	0.9316	0.8874	0.8600	**0.9326**	0.7305	0.9045	0.8847
	Sensitivity	0.9181	0.9074	0.8859	0.9482	0.8873	0.8939	**0.9837**
	Specificity	**0.9576**	0.8528	0.8177	0.9044	0.5158	0.9172	0.8426
	F1-Score	0.9424	0.9088	0.8883	0.9462	0.8038	0.9014	**0.9552**
	Precision	**0.9722**	0.9157	0.9005	0.9485	0.7585	0.9185	0.9089
W*k*NN	Accuracy	0.9453	0.8737	0.8463	**0.9663**	0.6942	0.7637	0.9626
	Sensitivity	**0.9420**	0.9103	0.8898	0.9772	0.8011	0.5401	0.9293
	Specificity	0.9517	0.8116	0.7705	0.9490	0.5533	**0.9961**	0.7514
	F1-Score	**0.9546**	0.9421	0.8798	0.9848	0.7625	0.6882	0.8896
	Precision	**1.0000**	0.9231	0.8794	**1.0000**	0.7549	0.9922	0.8648

**Table 8:** Using the *ID*_7_ dataset, results of the 3 classifiers for the given feature selection methods.

Models	Metrices	POS	Wilc	WSNR	mRMRe	Fisher	ROWSU	MW-RDS
RF	Accuracy	0.9552	0.9678	0.9821	**0.9952**	0.8964	0.9918	0.9903
	Sensitivity	0.9825	0.9877	0.9960	0.9975	0.9879	0.9924	**0.9980**
	Specificity	0.8000	0.8259	0.8664	0.9761	0.2179	**0.9913**	0.8460
	F1-Score	0.9739	0.9818	0.9899	**0.9973**	0.9437	0.9919	0.9945
	Precision	0.9655	0.9763	0.9842	**0.9972**	0.9043	0.9914	0.9910
SVM	Accuracy	0.8806	0.9237	0.9693	0.9722	0.8827	0.6802	**0.9800**
	Sensitivity	0.9825	0.9852	0.9809	0.9962	0.9949	0.6862	**0.9985**
	Specificity	0.3000	0.5055	0.8769	0.7883	0.0384	**0.9640**	0.6364
	F1-Score	0.9333	0.9580	0.9826	0.9846	0.9371	0.9826	**0.9891**
	Precision	0.8889	0.9341	**0.9846**	0.9738	0.8866	0.9661	0.9772
W*k*NN	Accuracy	0.8806	0.9582	0.9688	0.9933	0.8839	0.8385	**0.9939**
	Sensitivity	0.9649	0.9822	0.9876	**0.9990**	0.9748	0.6807	0.9984
	Specificity	0.4000	0.7910	0.8188	0.9490	0.2118	0.7033	**0.9784**
	F1-Score	0.9322	0.9852	0.9825	0.9967	0.9365	0.8085	**0.9985**
	Precision	0.9016	**1.0000**	0.9778	0.9828	0.9023	**1.0000**	0.9985

**Table 9:** Using the *ID*_8_ dataset, results of the 3 classifiers for the given feature selection methods.

Models	Metrices	POS	Wilc	WSNR	mRMRe	Fisher	ROWSU	MW-RDS
RF	Accuracy	0.7139	0.7689	0.7689	**0.9439**	0.7044	0.9040	0.7706
	Sensitivity	0.9160	0.9214	0.9214	0.9822	0.9364	0.8872	**1.0000**
	Specificity	0.1614	0.3632	0.3632	0.8337	0.0669	**0.9276**	0.1325
	F1-Score	0.8222	0.8546	0.8546	**0.9628**	0.8204	0.8987	0.9169
	Precision	0.7536	0.8087	0.8087	**0.9472**	0.7378	0.9183	0.7814
SVM	Accuracy	0.7350	0.7839	0.7839	0.9178	0.7356	**0.9333**	0.7683
	Sensitivity	0.9825	0.9517	0.9517	0.9738	0.9972	0.9979	**1.0000**
	Specificity	0.0558	0.3481	0.3481	0.7529	0.0000	**0.8705**	0.0300
	F1-Score	0.8432	0.8667	0.8667	0.9460	0.8450	0.9346	**0.9627**
	Precision	0.7437	0.8077	0.8077	0.9243	0.7373	0.8821	**0.9338**
W*k*NN	Accuracy	0.7144	0.7739	0.7739	**0.9461**	0.6594	0.5748	0.9133
	Sensitivity	0.8727	0.8626	0.8626	**0.9715**	0.8439	0.1513	0.9585
	Specificity	0.2849	0.5524	0.5524	0.864	0.1774	**1.0000**	0.1700
	F1-Score	0.8150	0.8051	0.8051	**0.9784**	0.7795	0.2955	0.8294
	Precision	0.7733	0.8824	0.8824	**1.0000**	0.7421	0.2367	0.7789

**Table 10:** Using the *ID*_9_ dataset, results of the 3 classifiers for the given feature selection methods.

Models	Metrices	POS	Wilc	WSNR	mRMRe	Fisher	ROWSU	MW-RDS
RF	Accuracy	**1.0000**	0.9380	0.9380	0.9380	0.9841	0.9040	0.9944
	Sensitivity	**1.0000**	0.3138	0.3138	0.3138	0.8375	0.8872	0.9283
	Specificity	**1.0000**	0.9864	0.9864	0.9864	0.9951	0.9276	**1.0000**
	F1-Score	**1.0000**	0.4115	0.4115	0.4115	0.8787	0.8987	0.9612
	Precision	**1.0000**	0.6602	0.6602	0.6602	0.9328	0.9183	**1.0000**
SVM	Accuracy	0.9627	0.9308	0.9308	0.9308	0.9726	0.9333	**0.9983**
	Sensitivity	0.4889	0.1453	0.1453	0.1453	0.6127	**0.9979**	0.5601
	Specificity	**1.0000**	0.9916	0.9916	0.9916	0.9999	0.8705	0.9997
	F1-Score	0.6467	0.2339	0.2224	0.2224	0.7527	**0.8669**	0.8124
	Precision	**1.0000**	0.6131	0.6351	0.6351	0.9987	0.8821	0.9964
W*k*NN	Accuracy	**0.9990**	0.9252	0.9252	0.9252	0.9822	0.5748	0.9984
	Sensitivity	**0.9941**	0.3126	0.3126	0.3126	0.8034	0.1513	0.6196
	Specificity	0.9993	0.9727	0.9727	0.9727	0.9958	0.2878	**0.9999**
	F1-Score	0.9924	0.2903	0.2903	0.2903	0.8045	0.1197	**1.0000**
	Precision	0.9911	0.2941	0.2941	0.2941	0.9000	0.1539	**0.9950**

For testing statistical significance, Table 11 presents the results of the Wilcoxon Rank-Sum test comparing MW-RDS against other methods. The findings reveal that the superiority of MW-RDS is also statistically significant.

**Table 11:** p-values by Wilcoson rank sum test comparing MW-RDS with feature selection methods across 9 datasets in terms classification accuracy. Statistically significance p-value (^*^p< 0.05, ^**^p< ^***^p<0.001) indicate that MW-RDS significantly outperforms the other method.

Datasets	Method	RF	SVM	WKNN
*ID*_1_	MW-RDS vs POS	0^***^	0^***^	0.000003 ^***^
	MW-RDS vs Wilcoxon	0^***^	0^***^	0.554
	MW-RDS vs WSNR	0^***^	0^***^	0^***^
	MW-RDS vs mRMRe	0.000002^***^	0^***^	1
	MW-RDS vs Fisher	0^***^	0^***^	0^***^
	MW-RDS vs ROWSU	0^***^	0^***^	0^***^
*ID*_2_	MW-RDS vs POS	0.0038^**^	0^***^	0.0146^*^
	MW-RDS vs Wilcoxon	0.0238 ^*^	0.000001 ^***^	0.4740
	MW-RDS vs WSNR	0.000002^***^	0^***^	0.000002^***^
	MW-RDS vs mRMRe	0.9950	0.0002^**^	0.9980
	MW-RDS vs Fisher	0^***^	0^***^	0^***^
	MW-RDS vs ROWSU	0.9990	0.0121^*^	1
*ID*_3_	MW-RDS vs POS	1	0.0766^*^	0^***^
	MW-RDS vs Wilcoxon	0^***^	0^***^	0^***^
	MW-RDS vs WSNR	0.0001^***^	0^***^	0^***^
	MW-RDS vs mRMRe	1	0.7180	1
	MW-RDS vs Fisher	0^***^	0^***^	0^***^
	MW-RDS vs ROWSU	0.9500	0.5950	0^***^
*ID*_4_	MW-RDS vs POS	1	0^***^	0^***^
	MW-RDS vs Wilcoxon	0.00001^***^	0^***^	0^***^
	MW-RDS vs WSNR	0.9560	0.0052^**^	0.8170
	MW-RDS vs mRMRe	0^***^	0^***^	1
	MW-RDS vs Fisher	0.1940	0^***^	0.0036^**^
	MW-RDS vs ROWSU	0^***^	0^***^	0^***^
*ID*_5_	MW-RDS vs POS	0.00005^***^	0^***^	0.231
	MW-RDS vs Wilcoxon	0^***^	0^***^	0.0367^*^
	MW-RDS vs WSNR	0^***^	0^***^	0.0136^*^
	MW-RDS vs mRMRe	0.960	0.00003 ^***^	1
	MW-RDS vs Fisher	0^***^	0^***^	0^***^
	MW-RDS vs ROWSU	0.857	0.0019^**^	0^***^
*ID*_6_	MW-RDS vs POS	1	1	0.0381^*^
	MW-RDS vs Wilcoxon	0.6250	0.9130	0^***^
	MW-RDS vs WSNR	0^***^	0^***^	0^***^
	MW-RDS vs mRMRe	1	1	0.917
	MW-RDS vs Fisher	0^***^	0^***^	0^***^
	MW-RDS vs ROWSU	0.992	0.975	0^***^
*ID*_7_	MW-RDS vs POS	0^***^	0^***^	0^***^
	MW-RDS vs Wilcoxon	0^***^	0^***^	0^***^
	MW-RDS vs WSNR	0^***^	0.0001^***^	0^***^
	MW-RDS vs mRMRe	0.910	0.0053^**^	0.1210
	MW-RDS vs Fisher	0^***^	0^***^	0^***^
	MW-RDS vs ROWSU	0.534	0^***^	0^***^
*ID*_8_	MW-RDS vs POS	0.00001^***^	0.0013^**^	0^***^
	MW-RDS vs Wilcoxon	0.670	0.957	0^***^
	MW-RDS vs WSNR	0.670	0.957	0^***^
	MW-RDS vs mRMRe	1	1	0.8950
	MW-RDS vs Fisher	0.00001^***^	0.0132^*^	0^***^
	MW-RDS vs ROWSU	1	1	0^***^
*ID*_9_	MW-RDS vs POS	1	0^***^	0.0015^**^
	MW-RDS vs Wilcoxon	0^***^	0^***^	0^***^
	MW-RDS vs WSNR	0^***^	0^***^	0^***^
	MW-RDS vs mRMRe	0^***^	0^***^	0^***^
	MW-RDS vs Fisher	0^***^	0^***^	0^***^
	MW-RDS vs ROWSU	0^***^	0^***^	0^***^

**Fig 2:**
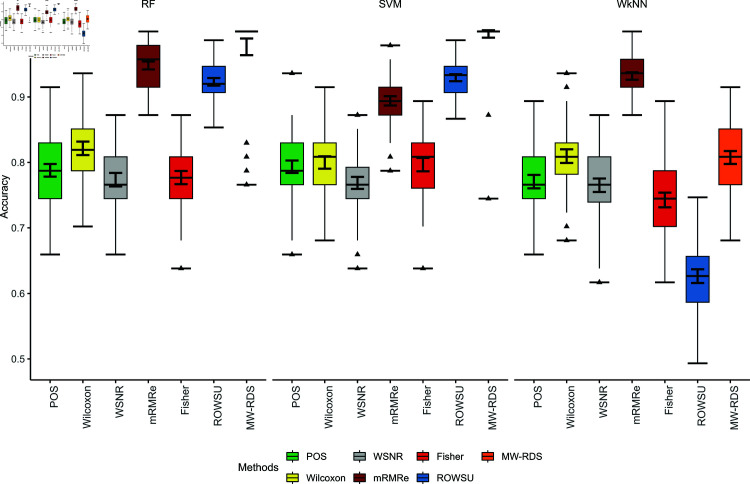
Boxplots of classification accuracies of the 3 classifiers for the given feature selection methods on *ID*_1_.

**Fig 3:**
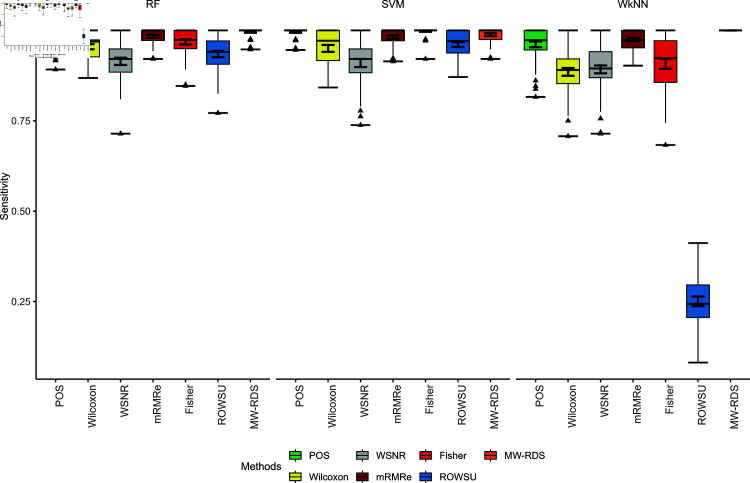
Boxplots of sensitivies of the 3 classifiers for the given feature selection methods on *ID*_1_.

**Fig 4:**
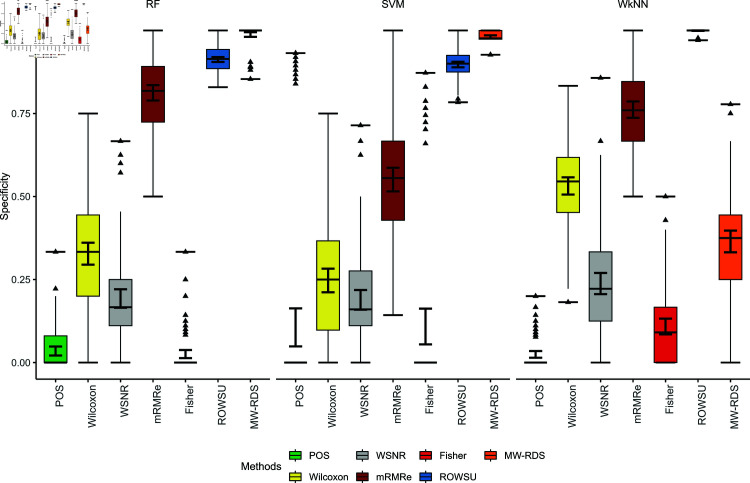
Boxplots of classification specificities of the 3 classifiers for the given feature selection methods on *ID*_1_.

**Fig 5:**
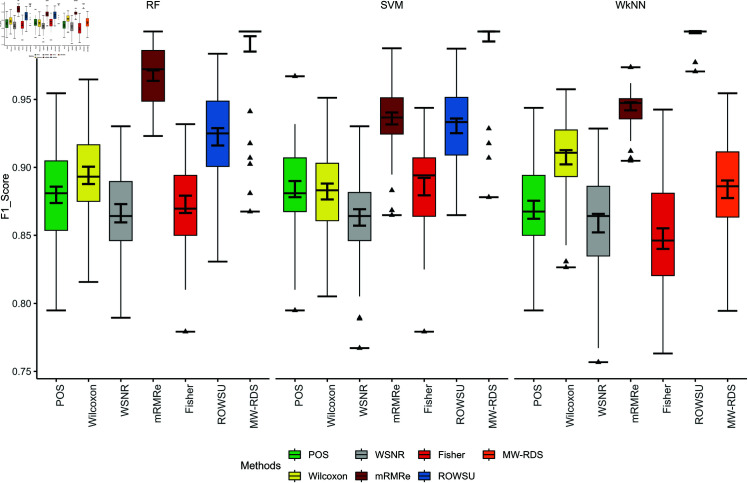
Boxplots of classification F1-scores of the 3 classifiers for the given feature selection methods on *ID*_1_.

**Fig 6:**
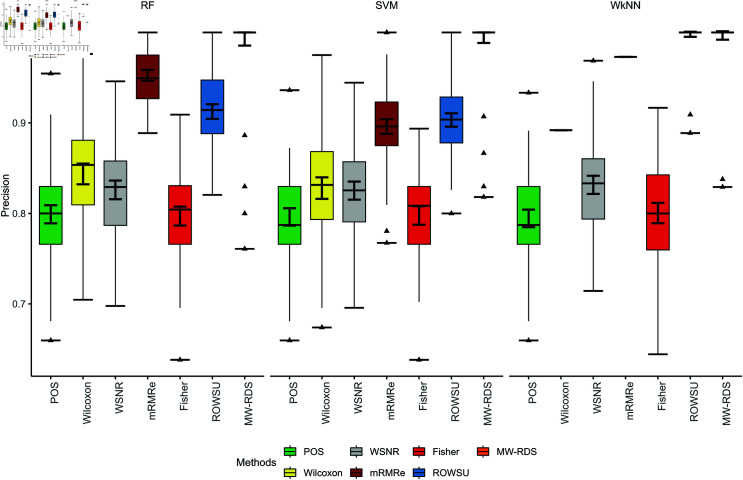
Boxplots of classification precisions of the 3 classifiers for the given feature selection methods on *ID*_1_.

The efficiency of the proposed method is further demonstrated through boxplots given in Figs 2, 3, 4, 5, and 6 for the various performance metrics on *ID*_1_. As shown in the boxplots, where the error bars denote mean±1.96SD, the proposed method exhibits significantly higher performance metrics with reduced variability relative to the competing methods, underscoring its robustness and superior effectiveness.

**Fig 7:**
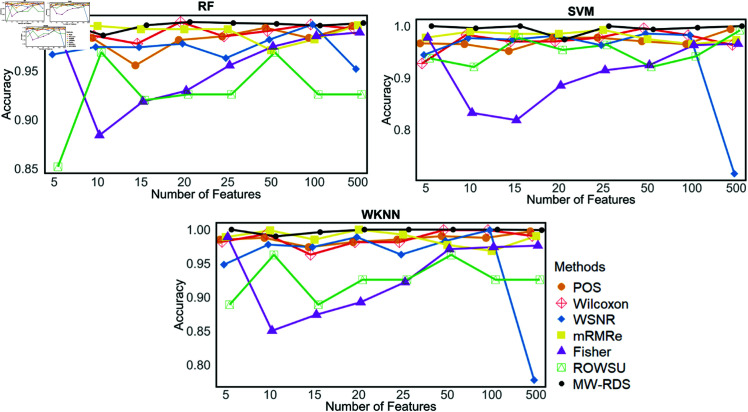
Plots of classification accuracies on *ID*_2_ for various numbers of selected features.

**Fig 8:**
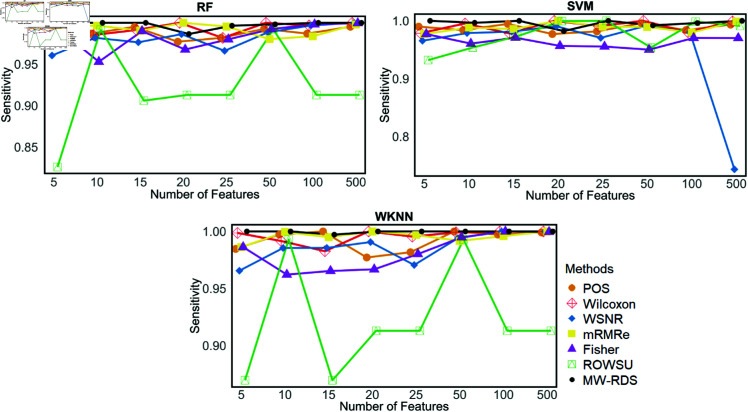
Plots of sensitivites on *ID*_2_ for various numbers of selected features.

**Fig 9:**
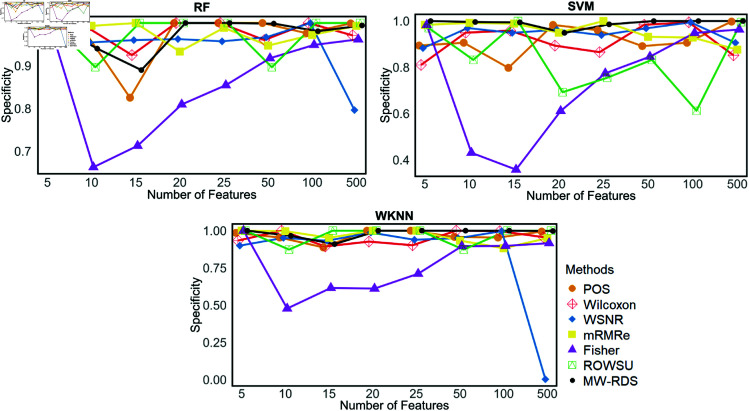
Plots of specificities on *ID*_2_ for various numbers of selected features.

**Fig 10:**
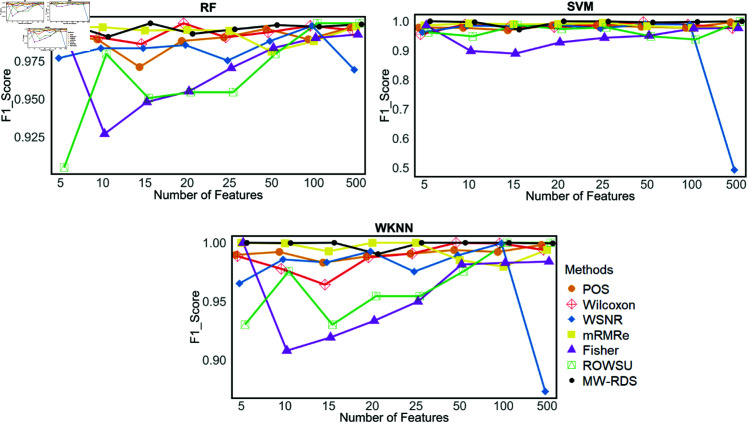
Plots of F1-scores on *ID*_2_ for various numbers of selected features.

**Fig 11:**
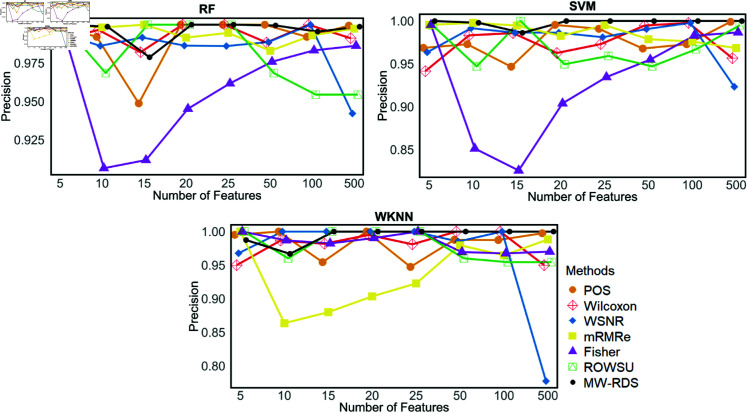
Plots of precisions on *ID*_2_ for various numbers of selected features.

Additionally, the metric plots given in Figs 7, 8, 9, 10, and 11 give a comparison of the proposed method with the other methods across various numbers of genes, i.e., 5, 10, 15, 20, 25, 50, 100, and 500. These plots highlight the consistent performance of MW-RDS against the others while selecting different number of genes. The results indicate that the proposed method exhibits greater stability compared to the other methods, even when the number of genes varies.

The primary aim of the study was to develop a gene selection method that enhances the classification performance of machine learning algorithms on imbalanced high-dimensional gene expression datasets. This study aims to assists readers interested in further exploring the biological significance of these selected genes. For example, the indices of selected genes are, G2599, G1036, G1495, G4303, G2722, G7747, G1830, G136, G3323, G9247, for the *ID*_1_ dataset while the indices of genes for *ID*_2_ are G4731, G7721, G10459, G6254, G4209, G5347, G5744, G6121, G10477, G10488 in order of their selection frequency among all the 500 runs. MW-RDS identified top 10 genes that give impressive 99.59% accuracy with SVM classifier for *ID*_1_ dataset. Similarly, the accuracy reached 97.66%, 99.62% using RF and SVM for *ID*_2_ dataset. For further reading on biological significance of the selected genes, readers are advised to see the work done in [46,47].

### 4.4 Simulation

This section presents two simulation scenarios to demonstrate the applicability of the proposed method. The first scenario highlights the effectiveness of MW-RDS in addressing imbalanced datasets, while the second scenario explores a data-generating environment where the proposed method might struggle.

To evaluate the performance of feature selection methods on imbalanced datasets, we simulate datasets with skewed class distributions, similar to the characteristics described in the benchmark analysis. The dataset contains κ=100 samples and 𝒳=5000 features. An imbalance ratio of 9:1 signifies that 90% of the observations belong to the majority class $\left|\kappa^{-}\right|$, and the remaining 10% represent the minority class $\left|\kappa^{+}\right|$. The exact number of majority/minority observations is computed using the following equations:

$$\kappa^{+}=\frac{\kappa \cdot \tau }{1+\tau },$$ (12)

$$\kappa^{-}=\kappa -\kappa^{+}.$$ (13)

Imbalance ratio (τ) is defined by the proportions of class distributions expressed in Eqs 11 and 13. The majority/minority class consists of $|\kappa^{-}|=90$, $|\kappa^{+}|=10$ observations of the total κ=100 for simulation. Using multivariate normal distribution,the feature matrix 𝒳 is generated to represent continuous features values as:

$$𝒳=\kappa (\mu_{𝒴},\Sigma_{𝒴}),$$ (14)

where, $\mu_{𝒴}$, $\sigma_{𝒴}$ are the mean vector and covariance matrix for target variable 𝒴, with 𝒴∈{+,−}} indicating the minority and majority observations, respectively. The amplification factor (τ) adjusts the contributions of the minority ${𝒳}^{+}$ and majority ${𝒳}^{-}$ class features, ensuring that the minority class influence is not overshadowed by the majority class. This methodology provides a robust framework for testing the performance of feature selection methods, including the proposed MW-RDS, under challenging and realistic imbalanced data conditions.

**Fig 12:**
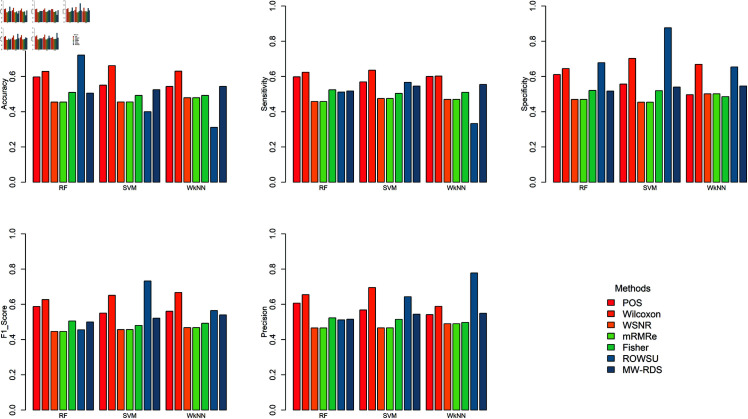
Barplots of results on the balanced simulated dataset based on 10 selected features.

**Fig 13:**
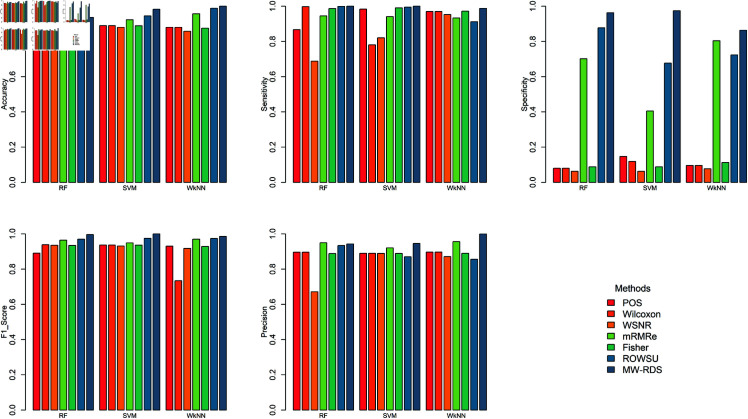
Barplots of the results on the imbalanced simulated dataset based on 10 selected features.

Fig 12 presents the comparison of the proposed and the other methods applied to the balanced simulated dataset, whereas, Fig 13 presents the results on the imbalance simulated data. While POS demonstrates strong performance under balanced scenario, it is worth highlighting the potential of MW-RDS, particularly in imbalanced scenarios given in Fig 13 showing its effectiveness by achieving higher accuracy via SVM, W*k*NN sensitivity via SVM, specificity via RF, SVM and W*k*NN, F1- score via RF, SVM and W*k* NN and precision via SVM and WkNN , which are crucial for accurately identifying minority class instances. This capability highlights the suitability of MW-RDS for addressing the challenges posed by imbalanced datasets, where maintaining equitable performance can be difficult for other methods. These results reflect the adaptability and robustness of MW-RDS, making it a valuable choice for diverse data distributions.

Table 12 summarizes the average time (in miliseconds) taken by each feature selection method for particular dataset. Methods, i.e., POS, WSNR and Fisher exhibit minimal computational cost due to their uni-variate design. In-contrast, MW-RDS requires slightly more time due to it enhanced design. Despite requiring slightly more time than basic filters, MW-RDS remains significantly faster than advanced method such as, wilcoxon, mRMRe and ROWSU. Although MW-RDS demands more computational cost to run, its notably improved classification performance makes it a worthwhile trade-off.

**Table 12:** Execution time (in miliseconds) of the feature selection methods for various number of features.

Features	POS	Wilcoxon	WSNR	mRMRe	Fisher	ROWSU	MWRDS
50	0.06	9826.81	567.30	6281.04	618.54	26891.84	3377.79
100	0.02	9753.18	574.42	17026.17	583.07	20905.67	3203.12
150	0.02	9786.82	571.88	35127.06	585.40	20091.18	3349.48
200	0.03	19055.11	568.05	59576.53	573.00	20810.78	3332.88
250	0.03	19056.43	571.61	90087.20	583.42	21335.45	3497.64
300	0.06	18882.10	577.25	83072.22	591.92	19703.40	3363.42
350	0.04	18831.37	579.28	107091.53	581.98	11856.37	3567.24
400	0.04	19086.21	578.38	134563.07	578.74	12037.87	3337.55
450	0.04	18795.63	578.01	205619.00	580.62	12781.28	3345.62
500	0.05	18870.65	550.60	245619.54	774.93	14569.49	3313.21

**Fig 14:**
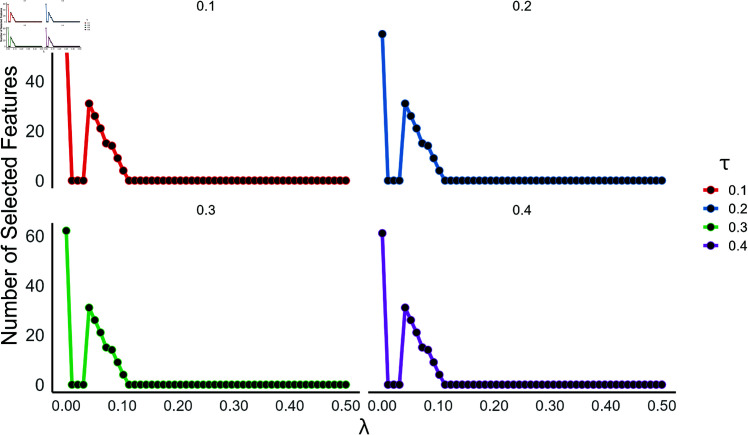
The effect of λ under different levels of minority amplification factor τ.

**Fig 15:**
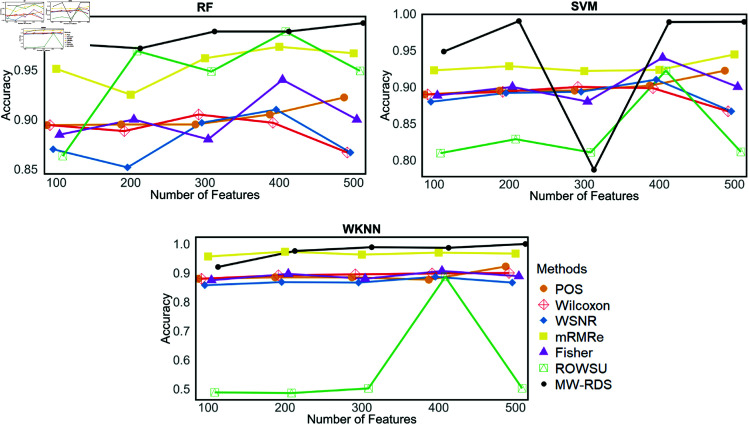
Classification accuracy of RF, SVM, and WKNN on 100–500 features selected by different methods for imbalanced simulated datasets.

**Fig 16:**
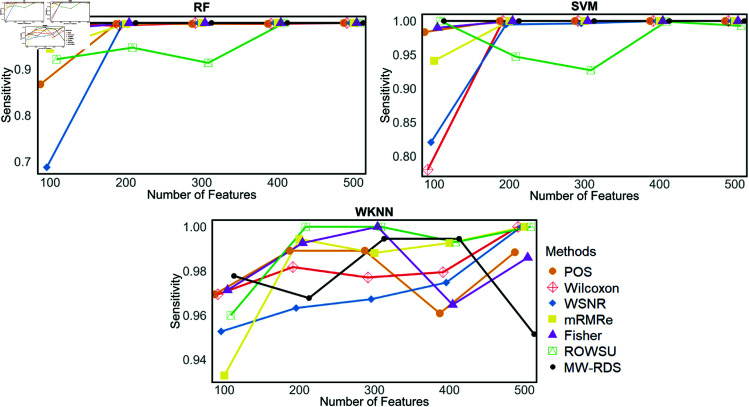
Sensitivity of RF, SVM, and WKNN on 100–500 features selected by different methods for imbalanced simulated datasets.

Furthermore, in terms of its sensitivity to choice of hyper-parameters, the number of features selected stayed almost the same by changing the level of minority amplification factor τ∈{0.10,0.20,0.30,0.40} across various setting of the regularization parameter λ. Fig 14 gives a clear demonstration the above on the simulated data. This behavior shows that MW-RDS is consistent across different τ-values, meaning that, minimal parameter fine-tuning may be sufficient. Ensuring efficiency for high dimensional imbalanced problem, the proposed method, MW-RDS time complexity is O(κ·p+plogp+dκ). Table 13 summarizes the performance of the proposed method compared to other feature selection methods on the top 50 features of the imbalanced simulated dataset. Classification was performed using RF, SVM, and WKNN models, and results for accuracy, sensitivity, specificity, F1-score, and precision are reported as mean±SD over 500 iterations. MW-RDS consistently achieved superior results in most of the cases. The performance of feature selection methods was further evaluated on the imbalanced simulated dataset using different number of features, 100,200,300,400,500. Figs 15 and 16 demonstrate that MW-RDS outperforms the other methods in terms of classification accuracy and sensitivity.

**Table 13:** Classification performance (accuracy, sensitivity, specificity, F1-score, and precision) based on 50 selected features, reported as mean±SD over 500 runs.

Method	Classifier	Accuracy $Mean ± SD)	Sensitivity $Mean ± SD)	Specificity $Mean ± SD)	F1_Score $Mean ± SD)	Precision $Mean ± SD)
MWRDS	RF	0.8523 ± 0.0547	0.7092 ± 0.0978	**0.9952 ± 0.0150**	**0.985 ± 0.0318**	**0.9210 ± 0.0672**
	SVM	**0.8285 ± 0.0502**	**0.9125 ± 0.0387**	**1 ± 0**	0.7846 ± 0.0708	**1 ± 0**
	WKNN	**0.8833 ± 0.0061**	**1 ± 0**	**1 ± 0**	**0.6495 ± 0.0533**	0.48333 ± 0.0615
ROWSU	RF	0.7233 ± 0.0685	**1 ± 0**	0.9171 ± 0.0378	0.3191 ± 0.1667	0.49 ± 0.2261
	SVM	0.3871 ± 0.1262	0.6507 ± 0.0980	**1 ± 0**	0.5856 ± 0.0899	0.6366 ± 0.1052
	WKNN	0.7200 ± 0.0612	**1 ± 0**	0.7723 ± 0.0361	0.5402± 0.0989	0.5026 ± 0.0782
Fisher	RF	0.6766 ± 0.0648	0.0983 ± 0.1086	0.9521 ± 0.0510	0.2313± 0.2139	0.0983 ± 0.1086
	SVM	0.6766 ± 0.0498	0.4400 ± 0.1758	0.9952 ± 0.0150	0.2626 ± 0.1286	0.7666 ± 0.0111
	WKNN	0.6466 ± 0.0877	0.2626 ± 0.1286	0.8335 ± 0.0777	0.7235 ± 0.0667	0.5436 ± 0.0899
mRMRe	RF	**0.8866 ± 0.0421**	0.7101 ± 0.1099	0.9727 ± 0.0374	**0.7916 ± 0.0722**	0.9196 ± 0.1133
	SVM	0.6933 ± 0.0644	0.0090 ± 0.0287	**1 ± 0**	0.0301 ± 0.0953	0.0090 ± 0.0287
	WKNN	0.8533 ± 0.0723	0.7533 ± 0.1409	0.9045 ± 0.0703	0.6380 ± 0.0558	**0.9 ± 0**
WSNR	RF	0.6500 ± 0.0849	0.1199 ± 0.1053	0.9104 ± 0.0693	0.2679 ± 0.1980	0.1199 ± 0.1053
	SVM	0.6233 ± 0.0861	0.1092 ± 0.1026	0.8756 ± 0.0990	0.2426 ± 0.1838	0.1092 ± 0.1026
	WKNN	0.6000 ± 0.0544	0.15841 ± 0.0966	0.8129 ± 0.0800	0.3140 ± 0.1712	0.2744 ± 0.1973
Wilcoxon	RF	0.7366 ± 0.0692	0.2442 ± 0.1359	0.9513 ± 0.0330	0.4473 ± 0.1884	0.6416 ± 0.2885
	SVM	0.7733 ± 0.0604	0.3724 ± 0.1386	0.9458 ± 0.0385	0.4875 ± 0.1481	0.7597 ± 0.1743
	WKNN	0.7466 ± 0.0632	0.4783 ± 0.1306	0.8672 ± 0.0543	0.3208 ± 0.0289	0.75 ± 0
POS	RF	0.7100 ± 0.0956	0.3004 ± 0.1928	0.9007 ± 0.0521	0.3548 ± 0.1768	0.5633 ± 0.2407
	SVM	0.7400 ± 0.0966	0.3004 ± 0.1662	0.9444 ± 0.0463	0.3961 ± 0.1808	0.695 ± 0.2564
	WKNN	0.6366 ± 0.0597	0.4728 ± 0.1443	0.7100 ± 0.0748	0.4263 ± 0.1353	0.4096 ± 0.1438

## 5 Conclusion

This study presented the Margin Weighted Robust Discriminant Score (MW-RDS), an innovative feature selection method designed to tackle the challenges of high-dimensional and imbalanced datasets.

In contrast to existing methods, MW-RDS presented a minority amplification factor, class-specific stability weights and margin weights from support vectors to confirm feature significance to minority class observations. Moreover, the use of $\ell_{1}$-regularization through the logistic function removed redundant features, resulting in extremely efficient feature set.

MW-RDS has been compared with feature selection methods, including Proportional Overlap Score (POS), Wilcoxon Rank-Sum Test (Wilcoxon), Weighted Signal-to-Noise Ratio (WSNR), ensemble Minimum Redundancy and Maximum Relevance (mRMRe), Fisher Score (Fisher) and robust weighted score for unbalanced data (ROWSU) on several benchmark gene expression imbalanced problems. Three classification models, i.e., Random Forest (RF), Support Vector Machines (SVM), and Weighted *k*-Nearest Neighbors (W*k*-NN) are used in terms of accuracy, sensitivity, specificity, F1-score, and precision to see the efficacy of the proposed method. MW-RDS consistently outperformed existing methods showing its ability to handle class imbalanced problem and achieve superior classification results.

Although MW-RDS involves a slightly higher computational cost as compared to some of the other feature selection methods, this trade-off has resulted in notably improved performance. Its robustness across various hyper-parameter settings further imply its effectiveness. Overall, MW-RDS provides a promising balance between efficiency and effectiveness for high-dimensional analysis with class imbalance.
